# Event-related potential (ERP) markers of 22q11.2 deletion syndrome and associated psychosis

**DOI:** 10.1186/s11689-023-09487-9

**Published:** 2023-06-16

**Authors:** Ana A. Francisco, John J. Foxe, Sophie Molholm

**Affiliations:** 1 Department of Pediatrics, The Cognitive Neurophysiology Laboratory, Albert Einstein College of Medicine, Bronx, NY USA; 2 Department of Neuroscience, Rose F. Kennedy Center, Albert Einstein College of Medicine, Bronx, NY USA; 3 Department of Neuroscience, The Frederick J. and Marion A, Schindler Cognitive Neurophysiology Laboratory, The Ernest J. Del Monde Institute for Neuroscience, University of Rochester, School of Medicine and Dentistry, Rochester, NY USA

**Keywords:** EEG, Velo-cardio-facial syndrome, DiGeorge syndrome, Schizophrenia, Sensory processing, Response inhibition, Error monitoring

## Abstract

22q11.2 deletion syndrome (22q11.2DS) is a multisystemic disorder characterized by a wide range of clinical features, ranging from life-threatening to less severe conditions. One-third of individuals with the deletion live with mild to moderate intellectual disability; approximately 60% meet criteria for at least one psychiatric condition.

22q11.2DS has become an important model for several medical, developmental, and psychiatric disorders. We have been particularly interested in understanding the risk for psychosis in this population: Approximately 30% of the individuals with the deletion go on to develop schizophrenia. The characterization of cognitive and neural differences between those individuals who develop schizophrenia and those who do not, despite being at genetic risk, holds important promise in what pertains to the clarification of paths to disease and to the development of tools for early identification and intervention.

Here, we review our previous event-related potential (ERP) findings as potential markers for 22q11.2DS and the associated risk for psychosis, while discussing others’ work. We focus on auditory processing (auditory-evoked potentials, auditory adaptation, and auditory sensory memory), visual processing (visual-evoked potentials and visual adaptation), and inhibition and error monitoring.

The findings discussed suggest basic mechanistic and disease process effects on neural processing in 22q11.2DS that are present in both early sensory and later cognitive processing, with possible implications for phenotype. In early sensory processes, both during auditory and visual processing, two mechanisms that impact neural responses in opposite ways seem to coexist—one related to the deletion, which increases brain responses; another linked to psychosis, decreasing neural activity. Later, higher-order cognitive processes may be equally relevant as markers for psychosis. More specifically, we argue that components related to error monitoring may hold particular promise in the study of risk for schizophrenia in the general population.

## Background

22q11.2 deletion syndrome (22q11.2DS), also identified as velo-cardio-facial or DiGeorge syndrome, occurs in approximately 1:1524 to 1:4000 live births [1, 2]. This mostly de novo deletion results from meiotic recombination events in four regions known as A-D low-copy repeats (LCR) on the long (q) arm of chromosome 22. About 85% of those living with 22q11.2DS present a deletion of the entire 2.5- to 3-Mb LCR A-D region; the remainder have smaller nested deletions within that region [3, 4]. There are 90 known or predicted genes present in the 3-Mb 22q11.2 locus that are hemizygously deleted [5]. Of those, about 90% have documented expression in the brain and may affect early neuronal migration and cortical development [5–7].

22q112.DS is a multisystemic disorder characterized by a wide range of clinical features, ranging from life-threatening to less severe conditions [8]. Common medical issues involve congenital heart defects, palatal abnormalities, immunodeficiency, hypocalcemia, genitourinary defects, and feeding and gastrointestinal problems [9, 10]. Cognitively, the majority of individuals with 22q11.2DS present an intellectual level in the borderline range (IQ scores between 70 and 84), and about one-third live with mild to moderate intellectual disability [11]. Intellectual ability appears to relate, in this population, to deletion size: Individuals with smaller (A-B) deletions have modestly higher IQ scores than those with larger (A-D) deletions [12]. Although the neurocognitive profile associated with the syndrome is quite variable between individuals, 22q11.2DS is characterized by overall deficits in executive function [13–16], nonverbal memory [17, 18], visuospatial [19–21] and visual-motor [22] processing, and working memory [23]. Individuals living with 22q11.2DS are also at an increased risk for developing psychiatric conditions: Approximately 60% meet criteria for at least one psychiatric diagnosis. Attention deficit with hyperactivity disorder (ADHD), autism spectrum disorder (ASD), anxiety and mood disorders, and psychotic disorders and schizophrenia have all been described in association with the syndrome [24–30]. Increased risk for psychiatric conditions may be related to specific cognitive trajectories in 22q11.2DS. While different trajectories have been identified in the syndrome—of relatively stable IQ, decline as a result of stagnation of cognitive development relative to increasing cognitive requirements, and absolute loss of cognitive abilities [31, 32]—the latter appears to be particularly associated with an increased risk for developing a psychiatric disorder [33].

In the past decade, 22q11.2DS has become an important model for several medical, developmental, and psychiatric disorders. Allowing for a better understanding of different conditions, the study of 22q11.2DS provides unique opportunities to clarify trajectories from risk to expression of disease. Consequently, the potential for the development of translational strategies and early interventions increases for both individuals with 22q11.2DS and those with associated features in the general population.

We have been particularly interested in understanding the risk for psychosis in this population. With 20 to 40% of individuals identified with the deletion going on to receive a formal diagnosis of schizophrenia [27–30], such risk is one of the most significant concerns for parents of children with 22q11.2DS. Importantly, considering the overwhelming burden of severe mental illnesses such as schizophrenia, clarifying paths to disease and developing tools for early identification prior to frank disease onset hold real promise for those living with this type of condition. Indeed, the detection of neural vulnerability prior to observable symptoms is crucial for the development of interventions focused on prevention rather than on treatment. Of note, idiopathic and 22q11.2DS-associated schizophrenia present similar clinical paths [33, 34] and clinical presentations [35] and high concordance of neuroanatomic correlates [36–41], which suggests that comparable neural changes could be explaining psychotic symptomatology in both populations. With approximately half of the adolescents with 22q11.2DS showing schizotypical traits and experiencing transient psychotic states [42], subthreshold psychotic symptoms appear, however, to present earlier in this group when compared to individuals in the general population who develop schizophrenia.

Given that all individuals with 22q11.2DS are at genetic risk for schizophrenia but not all develop the illness, a promising approach to understand markers of risk and disease is the comparison between those who develop psychotic symptoms and those who do not, despite being at risk. Our work suggests differences in behavior and brain responses between these 22q11.2DS subgroups. For instance, while individuals with the deletion but no psychotic symptoms were as fast as their age-matched control peers while pressing a mouse button during a go/no-go task, those with the deletion and psychotic symptoms were significantly slower [43]. Additionally, increased early evoked responses during auditory and visual tasks are observed in individuals with 22q11.2DS without psychotic symptoms, whereas those with the deletion and psychotic symptoms show reduced responses, quite similar to findings in individuals living with schizophrenia [44, 45].

Here, supported by findings from our work and contextualized by the extant literature, we consider the promise of event-related potential (ERP) biomarkers of risk for psychosis in 22q11.2DS, with the ultimate goal of generating new and critical questions regarding not only the true utility of such markers, but also the biology underlying risk of psychosis. Biomarkers, characteristics that are measured as indicators of typical or atypical biological processes or responses to an exposure or intervention [46], should be highly reproduceable, accessible measures with a sizeable signal-to-noise ratio that are modified in dynamic and reliable ways as the clinical condition progresses [47]. Electroencephalography (EEG), an easily deployed non-invasive method that provides information at the millisecond scale, allows one to probe the spatiotemporal dynamics of information processing in the brain. Because of its high temporal resolution, it permits the distinction between early sensory, sensory-perceptual, and later cognitive stages of processing [48, 50] and thus allows one to determine the stage at which information processing may be impaired. ERPs, the scalp recorded voltage fluctuations of the EEG signal that are time locked to a particular event of interest are seen as a set of positive and negative deflections in the evoked response. These evoked responses, or components, reflect activity within and across an often complex network of cortical regions [51, 52].

Considering the focus of ours and others’ work, this review covers (1) auditory processing (auditory-evoked potentials, auditory adaptation, and auditory sensory memory), (2) visual processing (visual-evoked potentials and visual adaptation), and (3) inhibition and error monitoring. Importantly, these processes have been comprehensively investigated in schizophrenia and have been discussed as potential endophenotypes for the condition.

## Auditory processing

### Auditory-evoked potentials

Auditory-evoked potentials (AEPs) are electrical brain responses that follow the presentation of an auditory stimulus. AEPs can be subdivided into three sequences of waves, reflecting activity at different levels of processing: (1) brain stem response, occurring within the initial 8–12 ms; (2) middle-latency sequence, resulting from activity in thalamic nuclei and neurons in the primary auditory cortex and occurring between 8 and 50 ms; (3) long-latency or cortical responses, which reflect activity in higher-order auditory and association cortices and generally occur between 50 and 300 ms [52]. Here, the focus is on the latter and, more specifically, on components indexing basic auditory processing and auditory sensory memory.

### Basic auditory processing

**Fig. 1:**
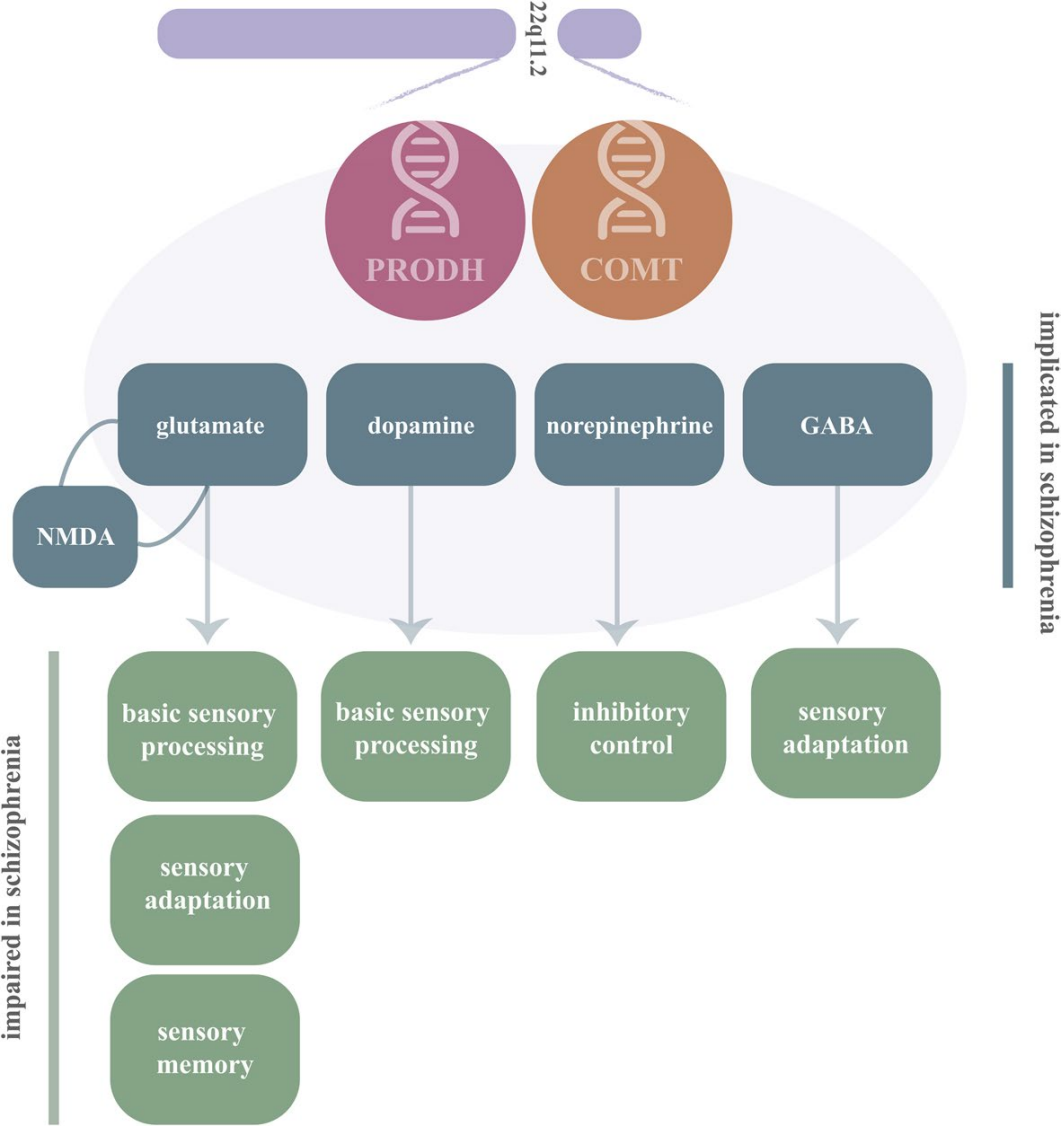
Representation of the implications of two of the genes in the 22q11.2 region implicated in schizophrenia, for the regulation of different neurotransmitters and specific measures of brain function

The auditory N1 is the first prominent negative AEP [50] and reflects neural activity generated in and around the primary auditory cortex [53]. In schizophrenia, N1 amplitudes are generally reduced [51, 54–57]. In contrast, increased N1 amplitudes appear to be observed in individuals with 22q11.2DS [58–60] and have been described in a 22q11.2DS mouse model [61]. Larger N1s have been associated with elevated activity in the anterior cingulate and dorsomedial frontal cortex [60] and associated with alterations in the cortical glutamate N-methyl-D-aspartate (NMDA) receptors [58, 62, 63] (see Fig. 1 for a simplified representation of the association between different neurotransmitters and brain mechanisms/processes addressed in the present review). Importantly, increased sensitivity to NMDA receptor antagonism has been described in a mouse model of 22q11.2DS [61] and elevated NMDA-receptor antibodies were found in a 19-year-old with the deletion and a history of cognitive decline and psychotic symptomatology [64].

**Fig. 2:**
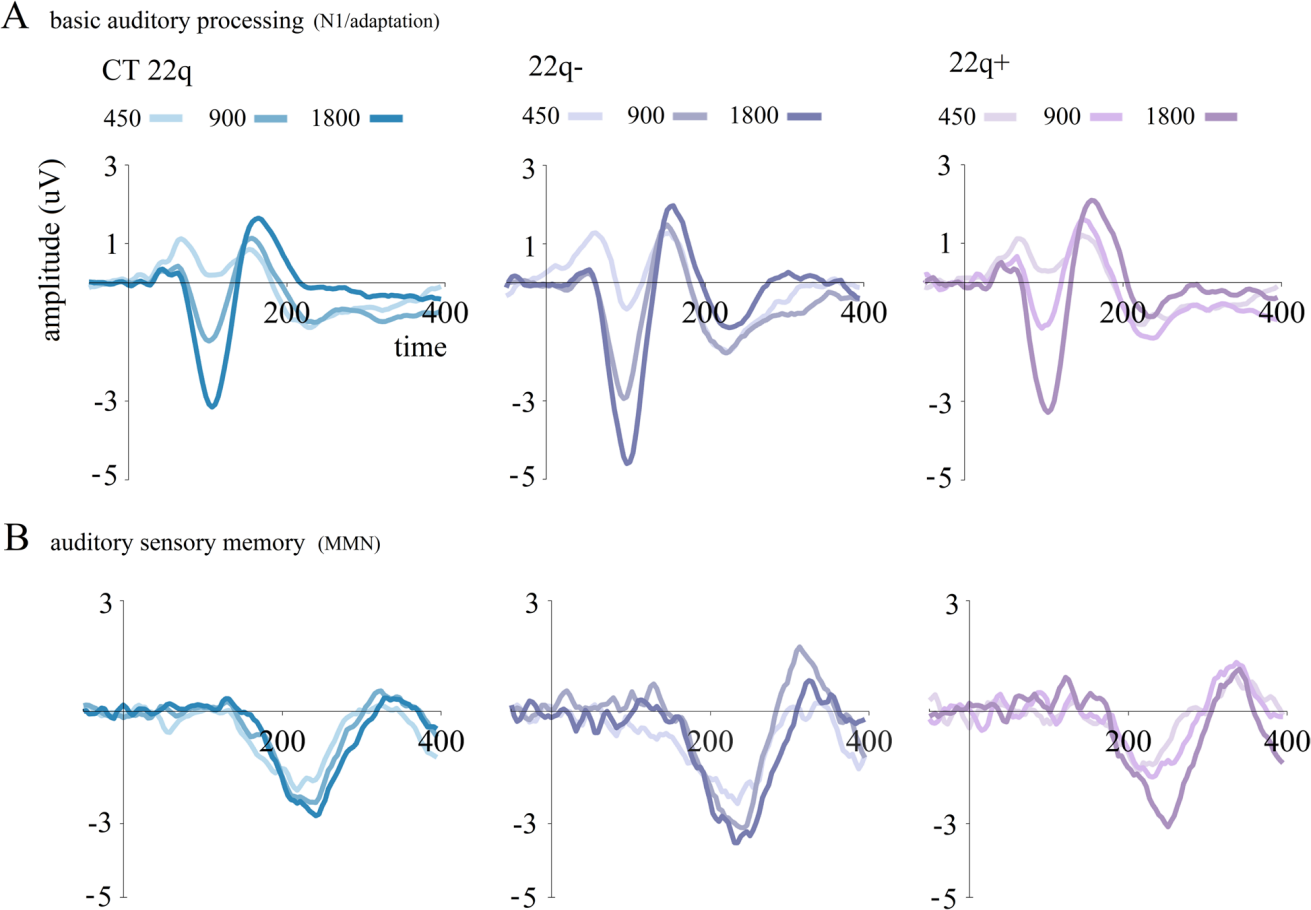
**A** Averaged ERPs (standard tones) per group (CT 22q, 22q − (without psychotic symptoms), 22q + (with psychotic symptoms)) and SOA (450, 900, 1800) at the fronto-central channel FCz. **B** Difference waves (deviants-standards) per group (CT 22q, 22q − , 22q +) and SOA (450, 900, 1800) at the fronto-central channel FCz. Adapted from [44]

Utilizing an oddball paradigm, we investigated basic auditory processing in individuals with 22q11.2DS. Unique to other studies on this topic [58, 59, 65–68], we focused on the potential differences between those with and without psychotic symptoms. We showed that while those without psychotic symptoms recapitulate the abovementioned increased N1 amplitudes, individuals with one or more psychotic symptoms showed reduced N1s (Fig. 2A), as is typically described in schizophrenia [51, 54–69]. Such reductions have been argued to index genetic risk for psychosis, as they have been described in first-degree relatives of individuals living with schizophrenia [57]. Our work, however, suggests otherwise, as those with the deletion and no psychotic symptoms (but still at-risk for psychosis) presented, here, increased amplitudes. Of note, these group differences were most evident in the differential response to different stimulation rates and appeared to be largely driven by longer stimulus onset asynchronies (SOA) conditions (900 and 1800 ms versus 450 ms) (see Fig. 2A), as discussed in the next section (Auditory Adaptation). We believe that there may be two mechanisms at work during early auditory sensory processing in 22q11.2DS: One related to the deletion resulting in the increased amplitudes observed in human and non-human animals; another associated with the presence of psychotic symptoms, which has as its outcome a decrease in brain responses. The auditory N1 may be modulated by both a deletion in chromosome 22 and the presence of psychotic symptomatology.

### Auditory adaptation

Sensory adaptation is an important property of sensory processing, as it appears to attenuate system redundancy [70–73]. Typically, in faster versus slower presentation rates of stimuli, amplitude reductions are observed, which can be explained by temporal limitations intrinsic to mechanistic brain response generation. That is, faster presentations do not allow for full response recovery, which results in decreased amplitudes [50, 74–78]. Other explanations of adaptation, related to priming and/or expectation have also been considered [79]. Though the nature of the neural mechanisms underlying adaptation is not fully understood, mechanisms such as neuronal refractoriness and presynaptic calcium influx have been implicated, as have NMDA-mediated glutamate transmission and GABAergic inhibition [80–83] (see Fig. 1).

In schizophrenia, altered adaptation to repetitive auditory stimuli has been reported [84–87], but this phenomenon has been sparsely studied in 22q11.2DS. The few studies published do not, moreover, provide a clear picture. While using sensory gating paradigms, some show intact [60, 88], others impaired [68] P50s. The P50 sensory gating is a measure of the suppression of the second P50 relative to the first P50 ERP in a paired-click paradigm assumed to reflect the ability to filter out repetitive, irrelevant stimuli and thus minimize information overload [89]. One study employing an oddball paradigm found reduced intrinsic connection within the right primary auditory cortex in a group of individuals with 22q112.DS but no psychotic symptoms. Such reduction was interpreted as suggestive of decreased adaptation in 22q11.2DS [59].

Our data, derived from a duration oddball paradigm with three different blocked SOAs (450, 900, and 1800 ms) supports “typical” adaptation in the N1 time window in 22q11.2DS, as can be appreciated in Fig. 2A. Indeed, regardless of the presence of psychotic symptoms, individuals with the deletion showed adaptation effects [44]. A distinction between those with and those without psychotic symptoms revealed, however, that, while the 22q11.2DS sub-group without psychotic symptoms showed increased adaptation effects, individuals with one or more symptoms showed decreased effects, compared to a control group. Interestingly, a closer look at our data revealed that it was the responses at the slower presentation rates that differed in the 22q11.2DS group, being larger in the 22q11.2DS group overall and in the sub-group without symptoms (Fig. 2A). These data seem to reiterate the presence of two different but important mechanisms in this early time window of sensory processing: One mechanism relating to 22q11.2DS and resulting in amplitude and adaptation effects increases, the other related to psychosis and causing overall amplitude reductions.

### Auditory sensory memory

Auditory sensory memory can be defined as a preattentive memory system that allows an individual to retain traces of sensory information after the termination of the original stimulus [90]. This very short memory can be assessed by means of the so-called mismatch negativity (MMN), generated during oddball paradigms. The MMN occurs when a repeating stimulus (the standard) in a sequence is replaced by a deviant stimulus. By violating the memory trace formed by the regularity of the consecutively presented standards, the deviant induces an MMN [91]. Typically ocurring 100 to 200 ms after the deviant, the MMN is thought to reflect largely preattentive neural processes underlying detection of a pattern violation and updating of a representation of a regularity in the auditory environment [92–94]. This component appears to be primarily mediated by the NMDA receptor [95] (Fig. 1).

Reduced MMN amplitudes have consistently been shown in schizophrenia (for reviews, see [96, 97]) in at-risk [98–102], recent onset [99, 100, 103–105], and chronic [99, 103, 106–113] stages of the condition, although findings are not always consistent and this is especially the case in first-episode and at-risk individuals where a number of negative findings have been reported [111, 114–117]. In 22q11.2DS, evidence is even less consistent (see, for a review, [118]). Whereas reduced pitch and duration [65] and frequency MMNs [58] have been reported, others have failed to show differences between individuals with 22q11.2DS and their control peers in frequency [59, 68], intensity, directionally, and duration deviants [68].

Such inconsistencies may again be a function of the phenotypic heterogeneity that is characteristic of 22q11.2DS. We had thus hypothesized that, as observed in the N1 time window, differences in MMN amplitudes would be found between those with and those without psychotic symptoms. It was therefore with surprise that we not only failed to observe differences between those two 22q11.2DS sub-groups, but we actually showed a slightly enhanced MMN in this clinical population [44] (see Fig. 2B). Given the well-established MMN reductions in schizophrenia and the weaker memory traces described in 22q11.2DS (see [119]), these findings were unexpected. That only a few of the individuals with 22q112.DS tested in this set of studies had a diagnosis of schizophrenia (the majority presented with subthreshold psychosis), and that our 22q11.2DS participants were younger than the typical individual with schizophrenia and have thus been living with symptoms for a significantly shorter period of time, likely explains the lack of effects seen in the MMN. Considering the inconsistent findings regarding MMN amplitudes in 22q11.2DS, the potential of this component as a biomarker of psychosis risk is not clear in this population.

## Visual processing

### Visual-evoked potentials

Visual-evoked potentials (VEPs) refer to electrical potentials recorded from the occipital and parietal scalp over the visual cortex. Here, the focus is on components evoked within the first 200 ms after stimulation.

### Basic visual processing

Early visual-evoked responses are generally reduced in schizophrenia (e.g., [120–131]). In 22q11.2DS, a combination of amplitude decreases and increases has been reported in response to visual stimulation [132, 133]. While reductions have been observed in P1 and N1 amplitudes, increased global amplitudes seem to emerge in time windows later than those traditionally associated with sensory-perceptual processing, around 250 ms [132]. These later increases in (frontal) activity in 22q11.2DS could reflect an increased recruitment of frontal regions to compensate for reduced activity in earlier processing in visual cortex [132]. Consistent with these EEG findings, there is evidence of atypical development and connectivity of occipital brain regions in this population. In a magnetic resonance imaging (MRI) study, widespread loss of white matter extending bilaterally in (among others) occipito-parietal regions was found in a small sample of adults with the deletion [134]. Given that glutamate is a crucial player in the neurotransmission within visual pathways, the proline dehydrogenase (PRODH) gene, a gene whose haploinsufficiency contributes to the clinical phenotype of 22q11.2DS, has been argued as a possible susceptibility gene for visual processing differences in this clinical population [133] (Fig. 1).

**Fig. 3:**
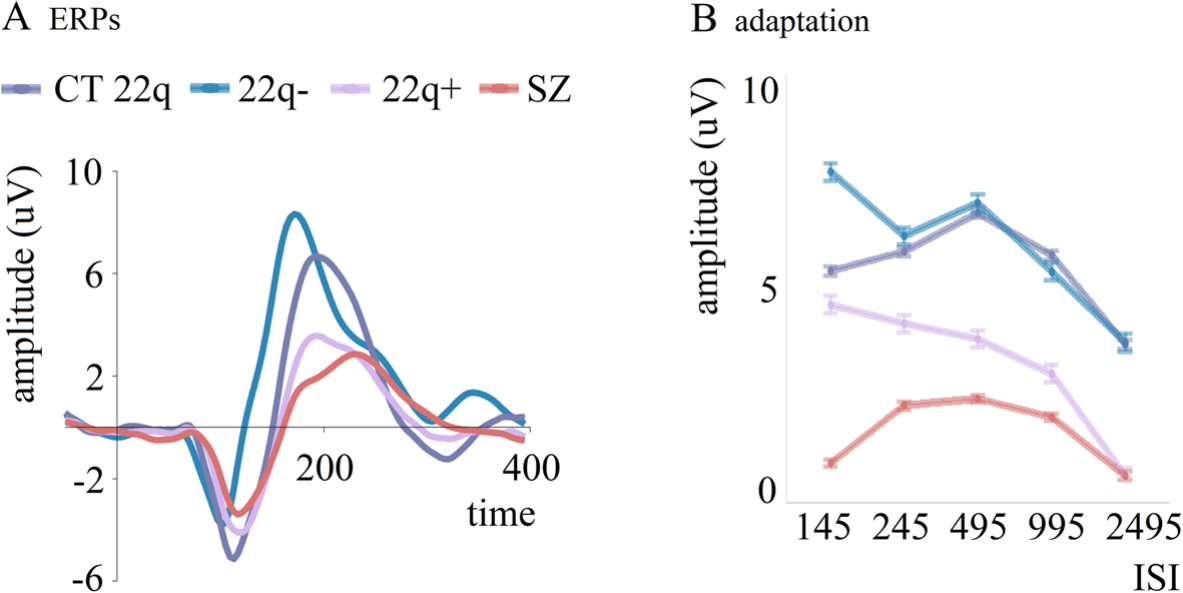
**A** Averaged ERPs per group (CT 22q, 22q − (without psychotic symptoms), 22q + (with psychotic symptoms), and SZ) at the occipital channel Oz, showing the average of all ISIs. **B** Curves representing visual adaptation effects between groups (CT 22q, 22q − , 22q + , and SZ) between 165 and 205 ms. Error bars represent standard errors of the mean. Adapted from [45]

Extending our investigation of early sensory processes as potential markers in 22q11.2DS and associated psychosis to visual processing, we utilized a visual adaptation paradigm previously used by our research group [120, 135] to question possible neural differences between those with and without psychotic symptoms. Much like what we found for auditory processing [44], we showed increased ERP amplitudes at around 100 ms for the group without psychotic symptoms, compared to those with psychotic symptoms and to age-matched controls [45] (Fig. 3A). Hence, the thesis of enhanced sensory processing in 22q11.2DS when in the absence of psychotic symptomatology appears to be likewise applicable to early visual processing. The groups also differed in a later (~ 200 ms) stage of processing, with those with the deletion and psychotic symptoms showing reduced amplitudes when compared to those without. Accordingly, while increased amplitudes in the earlier time window may reflect specific neurogenetic aspects associated with a deletion in chromosome 22, reduced amplitudes in the later window may be a marker of the presence and/or chronicity/severity of psychosis.

Alterations in NMDA receptors have been associated with increased early and decreased later neural responses [58, 62, 63, 136]. NMDA-related dysfunctional mechanisms may impact the modulation of sensory information reflected by early components and the efficiency of early attentional processes indexed by later components [136]. Atypical synaptic plasticity in early visual processing areas, resulting from both altered glutamate and dopamine levels [137, 138]—modulated by PRODH during early brain development [139]—could explain differences in visual sensory processing in 22q11.2DS (Fig. 1).

### Visual adaptation

Sensory adaptation is an important property of sensory processing [70–73], as indicated in the Auditory Adaptation section above. In schizophrenia, reduced visual adaption has also been reported [120, 140, 141], but see [84], though less consistently than in the auditory domain. To the best of our knowledge, no published study other than ours has addressed visual adaption in 22q11.2DS.

Utilizing a simple checkerboard adaptation task with five different interstimulus intervals (ISIs; 145, 245, 495, 995, and 2495 ms), we showed increased visual adaptation effects in individuals with the deletion, particularly in those without psychotic symptoms [45] (Fig. 3B). While larger adaptation effects could reflect better visual encoding efficiency and relate to enhanced visual short-term memory and attentional processes [142–144, 144], such a possibility seems exceedingly unlikely in 22q11.2DS, in which difficulties have been noted in visuospatial memory, attention, working memory, and other executive-type functions [17, 19, 44, 145–147]. As the neural mechanisms underlying adaptation are not well understood, the mechanistic implications of enlarged adaptation in 22q11.2DS remain relatively elusive. Interestingly, those with psychotic symptoms presented reduced adaptation effects, as expected in schizophrenia. In schizophrenia, reduced visual adaptation has been argued to contribute to the visual perception differences characteristic in psychosis [148]. Given that one proposed function of adaptation is that it improves discriminability of novel stimuli [149] and that one explanation for psychotic symptoms is the inappropriate attribution of salience to irrelevant stimuli, this hypothesis is particularly relevant for schizophrenia [150] and for those with 22q11.2DS and psychotic symptoms. These findings affirm, once again, that there are likely two processes at play here, one being the neurogenetic influence of a deletion in chromosome 22, and the other being the impact of psychosis on sensory processing. The similarity across modalities is, moreover, striking. Basic auditory and visual processing and adaptation fall into the basic and largely obligatory sensory processing domain. While they can be influenced by a participant’s state of alertness and by the presence or absence of directed attention, these influences are relatively small. Consequently, they are particularly appealing as candidate biomarkers.

## Inhibition and error monitoring

Inhibition and error monitoring are higher-order processes that fall in the realm of executive functioning. Briefly, executive function is an umbrella term that groups the set of cognitive control processes that govern goal-directed behavior and serve to optimize performance on complex tasks, allowing one to be flexible and to adapt to novel, ever-changing circumstances [151]. As these processes are crucial for function and interventional strategies can target their remediation, work on executive function components carries true potential for cognitive and daily function improvement.

### Inhibitory control

Inhibitory control, the process by which one suppresses a prepotent response that is irrelevant or inappropriate in a particular context, is essential for adjusting behavior dynamically with changing environmental contexts [152–154]. In schizophrenia, there is ample behavioral and EEG evidence of differences in inhibitory processes [155–160] and this has also been found in 22q11.2DS [161, 162]. Using a Go/No-Go EEG task (as in [161, 162], we asked whether neural differences related to inhibitory control in individuals with 22q11.2DS were modulated by the presence of psychotic symptoms. Our analyses focused on ERP components that are typically evoked during similar Go/No-Go tasks: The No-Go N2, a negative-going ERP component peaking between 200 and 300 ms and representing early, automatic inhibitory [163–166] and/or conflict detection processes [167–169], and the No-Go P3, a positive potential that peaks at about 300–500 ms, argued as a marker of response inhibition [170–174], stimulus evaluation [175–177] and adaptive, more effortful forms of control [165, 166, 178].

**Fig. 4:**
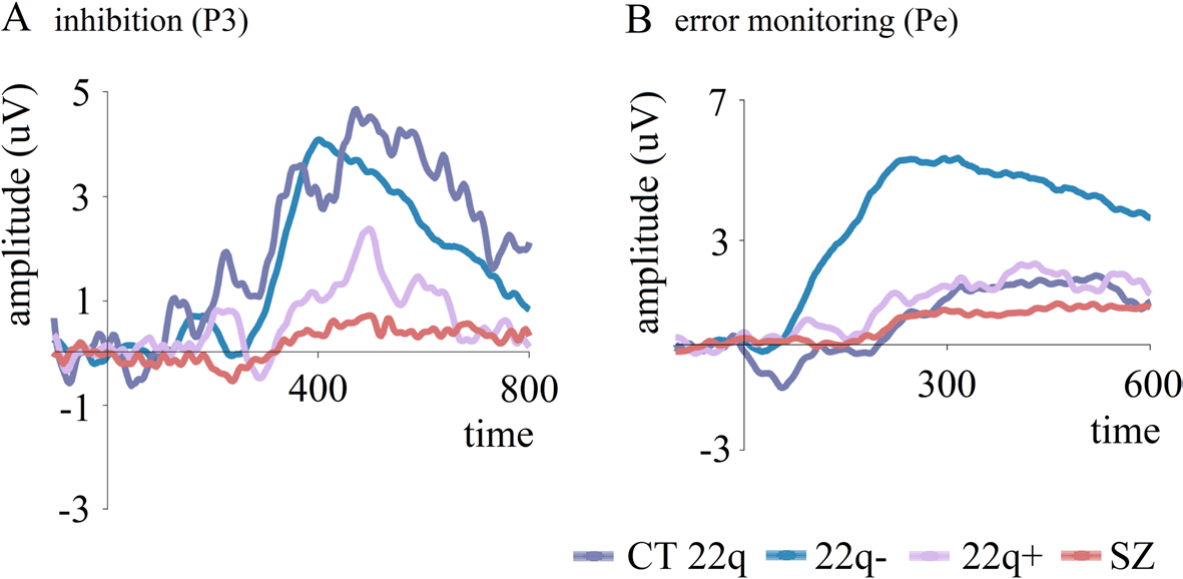
**A** No-Go P3 (difference waves: correct rejections – hits) per group (CT 22q, 22q − (without psychotic symptoms), 22q + (with psychotic symptoms), SZ) at the centro-parietal channel CPz. **B** Averaged ERPs depicting error-related positivity (Pe) per group at CPz. Adapted from [43]

As can be appreciated in Fig. 4A, our data revealed no differences between 22q11.2DS and controls or between those with and those without psychotic symptoms in the No-Go N2, possibly reflecting the lack of a clear N2 effect in either group. The N2 has been argued as a less reliable marker of response inhibition than the P3 [179–181]. Reductions in the No-Go P3 were, however, observed in 22q11.2DS, confirming possible difficulties in inhibitory-related processes (Fig. 4A). Additionally, those with smaller P3s performed worse in a standardized inhibition task, arguing for the No-Go P3 as reflecting the ability to inhibit a prepotent response. Interestingly, the No-Go P3 was only reduced in the 22q11.2DS group with psychotic symptoms, suggesting that this component could be a potential marker of the presence of psychotic symptoms [43] (Fig. 4A). Indeed, P3 reductions in schizophrenia [182] have been associated with disease severity, regardless of medication intake and task demands [183]. Amplitude reductions in this time window have, however, been shown in many other conditions such as chronic alcoholism [184] and ADHD [185], and thus, rather than conceiving a reduced P3 as a signature of schizophrenia-specific processes, one should probably conceptualize it as indexing general cognitive impairment in conditions characterized by inhibition deficits. Still, P3 could be useful in differentiating, within the 22q11.2DS population, those at higher risk to develop schizophrenia. P3 may reflect the activity of the neuromodulatory locus coeruleus (LC)–norepinephrine (NE) producing nucleus [186]. Norepinephrine dysfunction has been described in schizophrenia [187] and seems to be associated with its characteristic cognitive deficits [188]. COMT, encoding the protein catechol-O-methyltransferase responsible for degrading catecholamines such as norepinephrine (particularly in the prefrontal cortex), is a gene in the 22q11.2 region and is often thought of as a risk gene candidate for psychiatric disorders [8] (Fig. 1).

### Error monitoring

Error monitoring involves identification and correction of deviance from a correct response [189], and is required to achieve goal-directed behavior, to make appropriate adjustments to behavior, and to maintain task performance [190]. Two ERP components are often associated with error monitoring: The error-related negativity (ERN or Ne), a component occurring within 100 ms of an erroneous response, argued to reflect a mismatch between response selection and response execution [191, 192], but not remedial action [192]; and the error-related positivity (Pe), a component peaking between 200 and 500 ms post incorrect-response, which has been suggested to reflect conscious error processing or updating of error context [192, 193]. Reductions of Pe suggest a weakened (or even absent) sense of error awareness [194].

In schizophrenia, Ne is attenuated [195–199] and has been argued as a potentially important marker of risk, as such reductions have been shown in children with antecedents of schizophrenia [200], in high-risk individuals, early in the course of the disease, and in those living with chronic schizophrenia [201]. Though less consistently so, Pe reductions have likewise been reported in schizophrenia [201, 202], but see [197, 199, 203, 204]. To our knowledge, other than ours, no other study investigating these components in 22q11.2DS had been published previous to this review.

To investigate the potential of error monitoring-related components as markers of 22q11.2DS and/or the associated risk for psychosis, we focused on the neural activity following false alarms (i.e., responses to non-targets) in a Go/No-Go task [43] and compared individuals with 22q11.2DS with controls and individuals living with schizophrenia. As can be seen in Fig. 4B, for both Ne and Pe, our data revealed that not only did 22q11.2DS differ from controls, showing significantly reduced amplitudes, but no differences were seen between the two 22q11.2DS sub-groups. Ne and Pe may be potential markers of risk for schizophrenia, as all individuals with 22q11.2DS, regardless of the presence of psychotic symptoms, showed virtually absent componentry following the errors committed, a pattern that was no different to that which we observed in a group of individuals with chronic schizophrenia.

## Conclusion

**Fig. 5:**
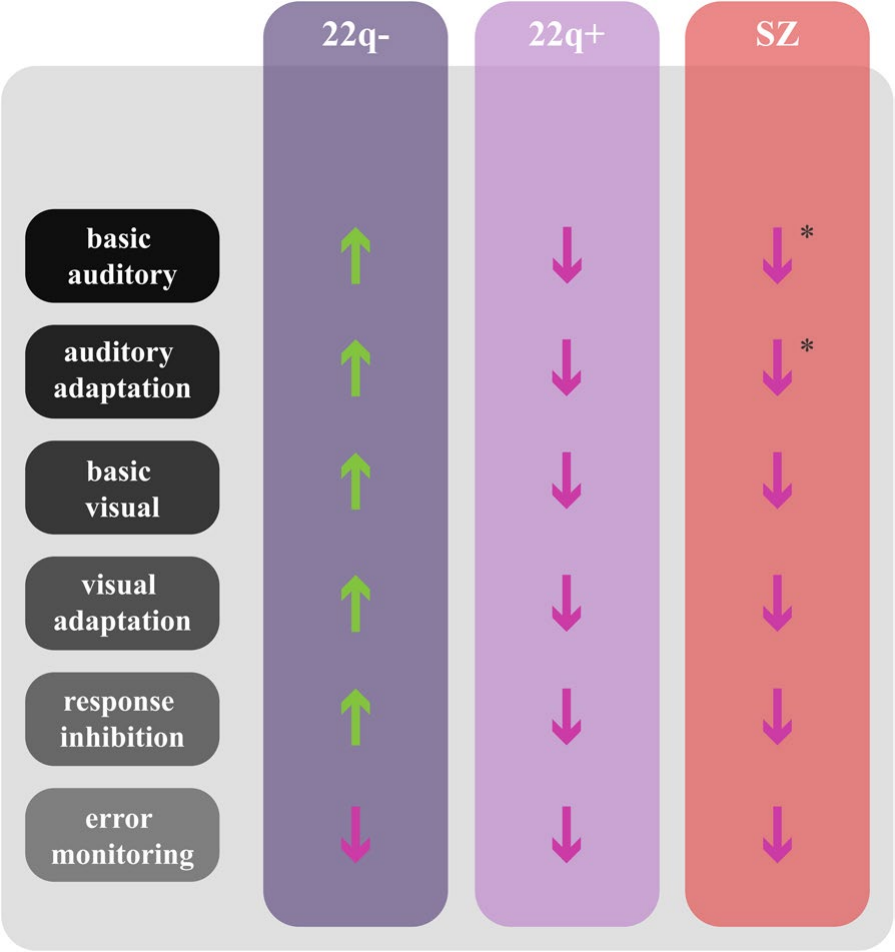
Graphic summary of findings, per process discussed and per group (22q11.2DS without psychotic symptoms: 22q − , 22q11.2DS with psychotic symptoms: 22q + , schizophrenia: SZ). Green, upward-pointing arrows represent increased response; Pink, downward pointing arrows represent decreased response. * Findings for basic auditory processing and auditory adaptation are based on the literature

Focusing on our previous work on auditory and visual sensory and higher-order cognitive (inhibition and error monitoring) processes in 22q11.DS, we reviewed and discussed those findings in the context of defining potential markers in this clinical population, particularly pertaining to risk for psychosis. As we focus on the mostly uncharted comparison between individuals with 22q11.2DS with and without psychotic symptoms and argue for what we believe is the potential impact of such comparison, not only for those living with the syndrome, but also for other at-risk groups, we believe that this review could generate new and critical questions with potential to advance the definition of meaningful markers of risk for psychosis. Figure 5 summarizes the findings discussed here.

Some work has been done in the past decade that approaches 22q11.2DS as a model for schizophrenia. Most studies have not, however, made a clear distinction or comparison between those individuals with the deletion and psychotic symptoms and those without psychotic symptomatology. The data discussed here clearly stress the importance of distinguishing between those with 22q11.2DS and psychotic symptoms and those without in the search for biomarkers. Indeed, in both auditory and visual sensory processing and executive function-related processes, we describe significant differences between those two sub-groups. In our opinion, it is in these differences that true understanding of pathways to disease may rest. Importantly, this approach can be generalized to any other associated features for which clear sub-groups can be formed. In characterizing phenotypically heterogenous syndromes such as 22q11.2DS, though it remains important to understand overall function, it is in the consideration of individual differences and sub-group trajectories that tangible potential for translational and interventional strategies may be found. For instance, it may be critical to consider individual differences in cognitive functioning and the extent to which those do or do not account for some of the findings reported in this review (of note, the 22q − and 22q + groups compared here did not differ in IQ scores). As argued for in schizophrenia [205], specific components or measures of cognitive function (e.g., verbal and non-verbal abilities) may serve as protective mechanisms in 22q11.2DS and should thus be leveraged.

The findings discussed here suggest basic mechanistic and disease process effects on neural processing in 22q11.2DS that are evident in early sensory and later cognitive processing, with possible implications for phenotype. As discussed in each section, most of the findings reported may be explained by processes related to the NMDA receptor complex and glutamatergic and GABAergic modulations, all possibly associated with PRODH and COMT, two of the genes in the region 11.2 in the long arm of chromosome 22 (see Fig. 1). The thorough investigation of NMDA-related function in 22q11.2DS, given its described associations with different components of sensory processing and seminal evidence of dysfunction in humans with the syndrome, animal models of 22q11.2DS, and in individuals living with schizophrenia, may hold particular promise in further characterizing the biology of (risk for) psychosis.

In summary, in early sensory processes, both during auditory and visual processing, two mechanisms that impact neural responses in opposite ways seem to coexist—one related to the deletion, which increases brain responses; another linked to psychosis, which decreases neural activity. Whether the amplitude enhancement in individuals with 22q11.2DS and no psychotic symptoms serves as a protective mechanism or is a mere consequence of the deletion, remains elusive. Additionally, it is unclear whether the decreased neural activity observed in those with psychotic symptoms is secondary to psychotic features or inherent to those who will develop them. Clarifying this matter is of incredible value to those living with a chromosome 22q11.2 deletion, but also to individuals in the general population who are at risk for developing schizophrenia. Indeed, it remains crucial to differentiate the global neural effects of living with psychosis from the biology contributing to psychosis itself. Longitudinal studies allowing for the study of the prodromal phase of psychosis could hold particular potential here.

We further show that later, higher-order cognitive processes may be equally relevant as markers for psychosis. More specifically, we argue that components related to error monitoring (Ne and Pe) may hold promise in the study of risk for schizophrenia in the general population. A more thorough, longitudinal investigation of these potential markers, the roles of genes in the 22q11.2 region in the conversion to psychosis, and NMDA-related dysfunctional mechanisms has potential to advance our knowledge about the contribution of specific neural and genetic processes (and of their interactions) to the onset of schizophrenia. Though the focus of the present manuscript is on the association between psychosis and a chromosome 22q11.2 deletion, other neurodevelopmental disorders have been observed in individuals living with the deletion. Future research should investigate if the presence of conditions such as ASD and ADHD, or symptoms related to these diagnoses, affect the pattern of findings reported here and take the complex inter-influences of clinical and cognitive variables into consideration.

## Data Availability

The datasets referred to in this manuscript are available from the corresponding authors on reasonable request. Analysis pipelines can be accessed at github.com/DouweHorsthuis.
